# Spinal Angiolipoma: A Rare Cause of Spinal Cord Compression

**DOI:** 10.7759/cureus.90273

**Published:** 2025-08-17

**Authors:** Elmehdi Hamidi, Kenza Jamai, Yassine Ait M'barek, Lamia Benantar, Khalid Aniba

**Affiliations:** 1 Neurological Surgery, Ibn Tofail Hospital, Mohammed VI University Hospital, Marrakech, MAR

**Keywords:** benign tumor, decompressive surgery, histological examination, spinal angiolipoma, spinal cord compression

## Abstract

Spinal angiolipomas (SALs) are uncommon benign tumors composed of mature adipocytes and abnormal blood vessels, with symptoms usually appearing gradually due to the progressive compression of the spinal cord. In this context, we report the case of a 34-year-old female patient with no significant medical history who presented with heaviness in both lower limbs and difficulty walking for the past six months. MRI showed an epidural posterior lesion extending from the first to the sixth thoracic vertebrae, strongly compressing the spinal cord, and surgical intervention for spinal cord decompression and total resection of the lesion was performed successfully. Histological examination revealed a lobulated lesion composed of two components: vascular and adipose, confirming the diagnosis. As SALs are rare benign tumors of the spinal cord characterized by a mixed proliferation of mature adipose tissue and abnormal vascular structures, their exact incidence remains unknown, although literature suggests a male predominance and a peak occurrence in adulthood. Clinical manifestations are typically progressive and depend on the level of spinal cord compression, making MRI the key diagnostic tool. Since treatment is primarily based on decompressive surgery, early diagnosis and intervention are essential. Although rare, SALs can be potentially debilitating due to their progressive compressive effects; therefore, MRI allows for a reliable diagnosis, and management relies on appropriate surgical excision. Long-term follow-up is crucial to prevent neurological complications.

## Introduction

Spinal angiolipomas (SALs) are uncommon benign tumors composed of mature adipocytes and abnormal blood vessels. They account for only 0.04-1.2% of all spinal axis tumors and about 2-3% of all extradural spinal tumors [1]. Given their rarity, these lesions are often overlooked in the differential diagnosis of space-occupying lesions within the spinal canal. The symptoms of SAL usually appear gradually because of the progressive compression of the cord. These masses are well-visualized on MRI. We report a case of dorsal epidural angiolipoma in a 34-year-old patient, manifested by paresthesia and difficulty walking. The outcome was favorable after early laminectomy and complete excision of the lesion.

## Case presentation

**Figure 1:**
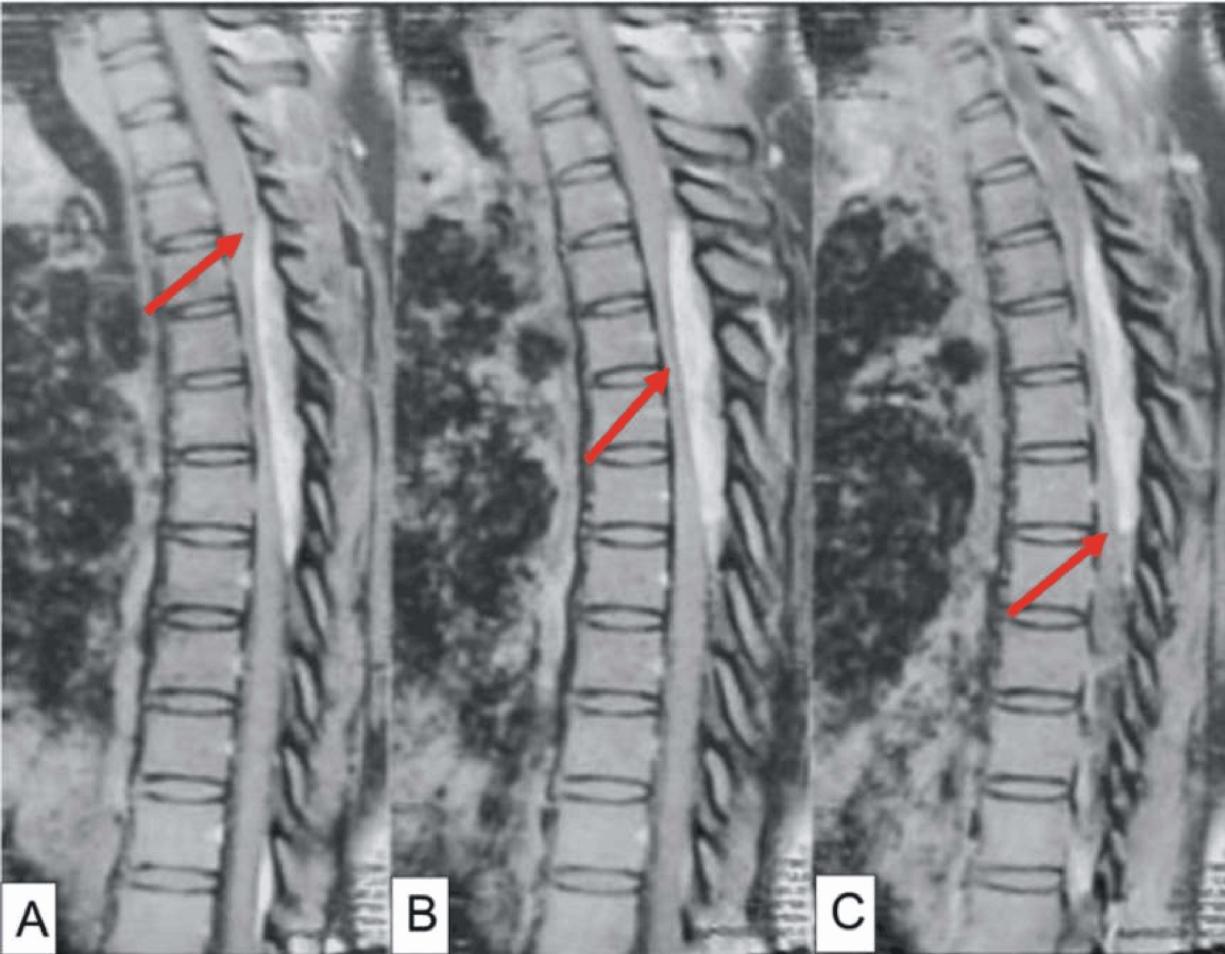
Dorsal MRI in T1 sagittal section showing the posterior epidural lesion (A) Dorsal MRI in T1 sagittal section showing the superior extent of the posterior epidural lesion. (B) Dorsal MRI in T1 sagittal section showing the posterior epidural lesion extension from D1 to D6, responsible for spinal cord compression. (C) Dorsal MRI in T1 sagittal section showing the inferior extent of the posterior epidural lesion

**Figure 2:**
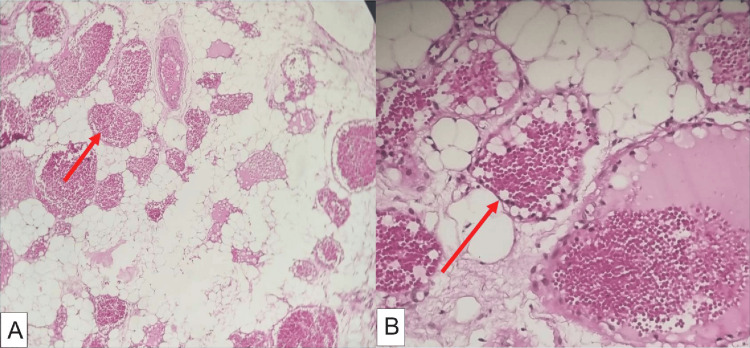
Histopathological examination of the tumor after resection (A) Image showing a biphasic proliferation of mature adipocyte lobules. (B) Image of a biphasic proliferation of mature adipocyte lobules, showing, when enlarged, a thin-walled cavernous vascular channels separation

A 34-year-old female patient with no significant medical history presents with heaviness in both lower limbs and difficulty walking for the past six months, without sphincter disturbances. Clinical examination reveals paraparesis with a pyramidal syndrome in both lower limbs and pain upon palpation of the spinous processes in the middle part of the dorsal spine. The MRI of the spinal cord shows an epidural posterior lesion extending from the first to the sixth thoracic vertebrae, which strongly compresses the spinal cord (Figure 1). A surgical intervention for spinal cord decompression with total resection of a process that was easily cleavable from the dura mater and bled upon contact was performed. The postoperative evolution was very favorable, with complete disappearance of paresthesias. The follow-up clinical examination at one month shows normal walking and total recovery of motor function. Histological examination revealed a lobulated lesion composed of two components: vascular and adipose, and a proliferation consisting of the juxtaposition of lobules of mature adipocytes and vascular cavities, concluding to a strictly benign angiomyolipoma (Figure 2).

## Discussion

SAL is a rare benign tumor of the spinal cord, characterized by a mixed proliferation of mature adipose tissue and abnormal vascular structures. It is distinct from intramedullary lipomas due to its well-developed vascular component [1].

This tumor is an exceptional entity among spinal tumors and is far more common in its subcutaneous location than within the spinal cord. Its exact incidence remains unknown, but literature reports suggest a male predominance and a peak occurrence in adulthood, typically between the ages of 20 and 60 [2].

SAL results from abnormal differentiation of embryonic tissues, involving both adipocyte proliferation and capillary hyperplasia. Some authors propose a congenital origin, while others suggest that tumor growth may be influenced by hormonal or ischemic factors [3]. Its slow progression explains the delayed diagnosis and the gradual onset of symptoms.

Clinical manifestations are progressive and depend on the level of spinal cord compression. Chronic back pain is often the first symptom, typically mechanical in nature but sometimes associated with an inflammatory component. As the disease advances, progressive neurological deficits may appear, including muscle weakness, sensory disturbances, and paresthesia. In more severe cases, a pyramidal syndrome can develop, characterized by hyperreflexia and spasticity, while sphincter dysfunction may occur in cases of significant compression [4].

MRI is the key diagnostic tool. T1-weighted sequences typically show a characteristic hyperintensity, with signal suppression in Fat-Sat sequences, confirming the adipose component. After contrast injection, moderate enhancement is usually observed, reflecting the lesion’s vascularization. A mass effect on the spinal cord is evident, but without bone invasion. Due to its distinctive radiological features, biopsy is rarely necessary [5].

Treatment is primarily based on decompressive surgery, aiming for total resection whenever possible. In cases where the angiolipoma is infiltrative or adherent to neurological structures, subtotal resection may be performed to minimize complications. In some instances, preoperative embolization may be considered to reduce intraoperative bleeding [6]. Radiotherapy is generally not indicated due to the tumor’s benign nature.

The prognosis is generally favorable following complete resection, with satisfactory functional recovery. However, when the lesion is infiltrative and resection is incomplete, persistent symptoms or slow recurrence may occur. Regular clinical and radiological follow-up is therefore essential, particularly through MRI, to detect any tumor recurrence or worsening spinal cord compression. The frequency of follow-up depends on the patient’s clinical evolution, but annual monitoring is typically recommended in the absence of recurrence [7].

## Conclusions

Although rare, SAL can be a potentially debilitating condition due to its progressive compressive effects. MRI allows for a reliable diagnosis, and management relies on appropriate surgical excision. Long-term follow-up is crucial to prevent neurological complications and ensure optimal patient care. A high index of suspicion is necessary, especially in patients presenting with gradual spinal cord symptoms. Early intervention can significantly improve outcomes and reduce the risk of permanent deficits. Continued clinical awareness is important to guide timely diagnosis and effective treatment.
